# Coupled Adsorption and Biodegradation of Trichloroethylene on Biochar from Pine Wood Wastes: A Combined Approach for a Sustainable Bioremediation Strategy

**DOI:** 10.3390/microorganisms10010101

**Published:** 2022-01-04

**Authors:** Marta M. Rossi, Bruna Matturro, Neda Amanat, Simona Rossetti, Marco Petrangeli Papini

**Affiliations:** 1 Department of Chemistry, Sapienza University of Rome, Piazzale Aldo Moro 5, 00185 Rome, Italy; neda.amanat@uniroma1.it (N.A.); marco.petrangelipapini@uniroma1.it (M.P.P.); 2 Water Research Institute (IRSA—CNR), Via Salaria km 29.300, 00015 Monterotondo, Italy; matturro@irsa.cnr.it (B.M.); simona.rossetti@irsa.cnr.it (S.R.)

**Keywords:** bioremediation, adsorption, biochar, biodegradation, reductive dechlorination, *Dehalococcoides mccartyi*

## Abstract

Towards chlorinated solvents, the effectiveness of the remediation strategy can be improved by combining a biological approach (e.g., anaerobic reductive dechlorination) with chemical/physical treatments (e.g., adsorption). A coupled adsorption and biodegradation (CAB) process for trichloroethylene (TCE) removal is proposed in a biofilm–biochar reactor (BBR) to assess whether biochar from pine wood (PWB) can support a dechlorinating biofilm by combining the TCE (100 µM) adsorption. The BBR operated for eight months in parallel with a biofilm reactor (BR)—no PWB (biological process alone), and with an abiotic biochar reactor (ABR)—no dechlorinating biofilm (only an adsorption mechanism). Two flow rates were investigated. Compared to the BR, which resulted in a TCE removal of 86.9 ± 11.9% and 78.73 ± 19.79%, the BBR demonstrated that PWB effectively adsorbs TCE and slows down the release of its intermediates. The elimination of TCE was quantitative, with 99.61 ± 0.79% and 99.87 ± 0.51% TCE removal. Interestingly, the biomarker of the reductive dechlorination process, *Dehalococcoides mccartyi*, was found in the BRR (9.2 × 10^5^ 16S rRNA gene copies/g), together with the specific genes *tceA*, *bvcA*, and *vcrA* (8.16 × 10^6^, 1.28 × 10^5^, and 8.01 × 10^3^ gene copies/g, respectively). This study suggests the feasibility of biochar to support the reductive dechlorination of *D. mccartyi*, opening new frontiers for field-scale applications.

## 1. Introduction

Chlorinated aliphatic hydrocarbons (CAHs) are some of the most common soil and groundwater contaminants, due to their chemical properties, production, and cost-effective uses [1,2]. Leaks, spills, and the storage of these solvents in the subsoil have caused the contamination of several environmental matrices [3]. By the late 1960s, the public became more aware of environmental issues, such that perchloroethylene (PCE), trichloroethylene (TCE), and tetrachloroethane (TeCA) were identified as harmful pollutants [4]. Years of field experience, based on pump-and-treat, have shown that this technology achieves a significant reduction in CAH concentrations in aquifers. However, the adsorbed mass of the solvents may persist in the aquitard and less permeable areas, acting as a secondary source of contamination [2,5]. Nowadays, more sustainable and less energy-intense technologies are required to address CAH contamination [6]. For instance, numerous advantages make in-situ bioremediation technologies an attractive and environmentally friendly choice [7]. From this perspective, reductive dechlorination (RD) should be considered a valid biological pathway for removing CAHs from the subsurface environment [8,9]. In particular, highly chlorinated compounds, i.e., PCE, can be biologically reduced by anaerobic organohalide respiring bacteria (OHRB) in the presence of an electron donor, including fermentable organic substrates or direct hydrogen [10,11]. Notably, the step-by-step reaction involves a progressive substitution of chlorides in such order: PCE, TCE, cis-DCE, VC, and ethylene. Among the OHRB, *Dehalococcoides mccartyi* is the primary biomarker of the RD process, which reduces PCE up to the nontoxic ethylene. Redox reactions are catalyzed by the enzymatic activity of reductive dehalogenases, including TceA, BvcA, and VcrA, characteristic of various *D. mccartyi* strains involved in different steps of the reductive dechlorination process [12].

The main objections of the RD strategy concern the bioavailability of the substrate (added by injection), and the time required to terminate the intervention (months to years to implement) [4,13]. To assess whether in situ bioremediation might be a feasible option for CAH-source-zone degradation, a detailed characterization of the site and feasibility studies (combining laboratory studies with field activities) must be performed [14,15]. Subsequently, to reduce the time needed to achieve remediation goals and overcome bioremediation limitations, a recommended strategy may be the combination of several synergistic technologies [16]. Indeed, the combination of abiotic degradation, e.g., by chemical reduction with zero-valent iron (ZVI), and the stimulation of biological metabolism is a diffused approach [17,18,19,20]. With this purpose, the immobilization of the contaminant by adsorption and the addition of an electron donor gave excellent results in a field-scale application [21]. To date, the available commercial products generally enhance and optimize activated carbon, using it as an adsorbent and a biofilm carrier [22,23], since the process seems to improve both the biodegradation and the regeneration of the material [24,25,26,27]. At the same time, scientific interest in alternative carbonaceous materials has increased, notably in residues from biomass heat treatment, i.e., biochar. Since biochar is a byproduct of pyrolysis/gasification of waste biomass, its production and application have been extensively increased worldwide [28,29]. Biochar is well known to improve soil quality precisely because of the interactions between the surface and nutrients [30], and recently, many studies aimed to demonstrate its interaction with the microbial communities of the subsoil [31,32,33,34]. The general mechanism is to immobilize different classes of pollutants, thanks to the carbon structure and functional groups present on the surface [35,36], resulting in the contaminants being ready for biodegradation [37]. These important effects should also be linked to the electron transfer capacity of biochar, which may be responsible for the degradation of several pollutants [38,39,40,41]. These premises explain why recent studies point to biochar as a possible support for the growth of specific bacterial consortia (as a biofilm carrier [42]) and for enhancing biodegradation of pollutants [25,43,44]. Recently the simultaneous adsorption and biodegradation of TCE have been studied on herbal pomace and spruce biochar [45]. In this research, pine wood biochar (PWB) was used as a growth medium for a dechlorinating biofilm to remove TCE from an aqueous solution. The same PWB was previously characterized from a textural and adsorption capacity point of view [46,47]. The degradation of dissolved TCE by coupled adsorption and biodegradation (CAB) was proposed in a fixed-bed reactor filled with PWB and previously inoculated with a TCE-to-ethylene *D. mccartyi*-enriched culture. The experiment was carried out in parallel with a reactor without PWB, to compare the behavior with and without the adsorption material. Two different flow rates were studied to investigate the RD-intermediate formation, depending on the hydraulic residence time (HRT). The reactors operated in parallel for eight months. To determine differences in the biofilm developed on the PWB reactive zone, *D. mccartyi* abundance (i.e., 16S rRNA and reductive dehalogenase genes) was monitored in the inoculum and the reactor at the end of the experiment. This study aimed to evaluate the performance of the CAB process with regards to the effectiveness of the biochar in sustaining the biological dechlorination, as well as monitoring the occurrence of the main dechlorinating microorganism *D. mccartyi*.

## 2. Materials and Methods

### 2.1. Fixed-Bed Reactor’s Setup

Two fixed-bed reactors were realized in plexiglass columns (23 cm length × 2.6 cm diameter). The reference reactor for the RD mechanism alone was packed with silica sand and is here identified as the “biofilm reactor” (BR). On the other hand, the second reactor, named the “biofilm–biochar reactor” (BBR), was filled with a mixture of silica sand and PWB (4% *w*/*w*, corresponding to 6 g weighed), to realize a reactive adsorption zone of 18 lengths and to simulate the coupled adsorption and biodegradation (CAB) mechanism. The biological mechanism was ensured by injecting a specific dechlorinating biomass that will be described below. A third abiotic reactor, which we will refer to as the “abiotic biochar reactor” (ABS), was prepared similarly to the BBR to observe the behavior of the TCE adsorption mechanism alone on PWB, thus, with no dechlorinating biomass.

**Figure 1:**
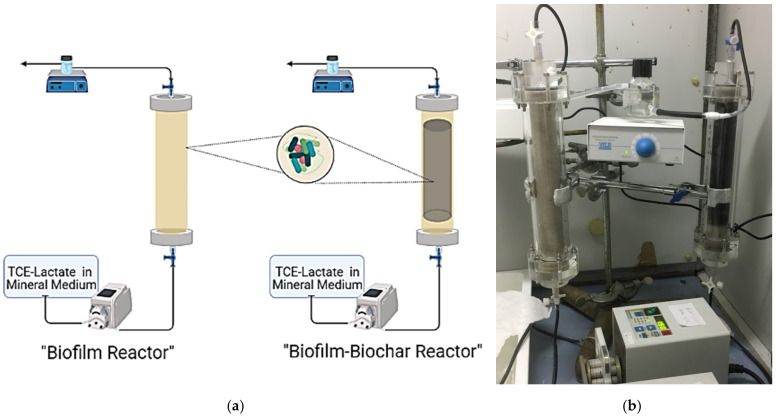
(**a**) BR and BBR setup; (**b**) a photograph of the BR (on the left) and BBR (on the right).

An Ismatec (Veneto, Italy) multichannel peristaltic pump was used to feed the reactors from the bottom (to eliminate air bubbles) and Viton tubing connected the systems. A three-way valve was placed at the entrance and exit. The outlet current was collected in a sampling cell, hermetically sealed with a cap, and magnetically stirred (micro stirrer VELP Scientifica, Monza and Brianza, Italy), from which it was possible to sample the gas phase for the CAH analysis (see Figure 1a,b).

Before dechlorinating the biomass addition, a tracer test was performed with KBr (100 mg/L ${\mathrm{Br}}^{-}$) and NaCl (0.02 M), for the BR and BBR respectively. Porosity and bed volume (BV) was calculated as described in Rossi et al. (2021) [46]. The flow rate was measured daily; hence, Table 1 shows the average values over the investigated period and the parameters consequently calculated. Afterwards, an active dechlorinating culture (100 mL) was injected into the BR and BBR by the inlet three-way valve and recirculated for at least 48 h for the biofilm’s stabilization. The feeding solution was prepared in Tedlar^®^ bags (Supelco, Bellefonte, PA, USA) to avoid headspace formation. The solution consisted of anaerobic mineral medium (flushed with N_2_/CO_2_ gas mixture), TCE (0.1 mM), and lactate (0.2 mM). The initial concentration (TCE IN) was verified by periodically sampling from the Tedlar^®^ bag’s septum. All of the reactors operated at room temperature (15–22 °C). During the COVID-19 emergency in the city of Rome (lockdown from March to May 2020), the reactors were left at the minimum flow rate (between 0.22 and 0.28 L/d). Testing of the ABR was conducted under HRT equal to 5 h and ended when the output concentration was about equal to the input concentration.

### 2.2. Pine Wood Biochar (PWB)

The PWB was used as filling material for the BBR. It was obtained from a V 3.90 Burkhardt ECO 180 HG wood gas generator as a thermal and electrical plant (Plößberg bei Tirschenreuth, Germany) at about 850 °C [47]. PWB has a high specific surface area (343 ± 2 m^2^/g), a total pore volume of 0.383 cm^3^/g (0.136 cm^3^/g due to micropores, and 6.6 Å of the distance between carbon planes), a pore size distribution in the 20–100 Å range [47], and a high percentage of carbon (95.84% wt) [46]. The adsorption capacity for specific contaminant targets (toluene and polycyclic aliphatic hydrocarbons) was demonstrated in previous studies [47,48]. More recently, as a chlorinated solvent target, the TCE removal capacity was investigated by Rossi and coworkers (2021), both in batch and column configuration and compared with biochar from other feedstocks and treatments. The PWB showed rapid kinetic removal, with a pseudo-second-order kinetic constant of 0.0053 (g/(mg/min)), reaching high amounts of adsorbed contaminant per gram of material. Based on the Langmuir isotherm model, the maximum amount adsorbed was 109.41 ± 5.62 mg TCE/g PWB, within the range of concentration of 10–120 mg/L. The PWB column (with 2 g of PWB in the reactive bed) removed 16.6 mg of TCE per gram of PWB (feeding solution contaminated with TCE at 0.04 mM) [46].

### 2.3. TCE-to-Ethylene Culture

The TCE-to-ethylene dechlorinating enrichment culture used as inoculum for the BR and BBR was derived from a PCE-dechlorinating consortium, where *D. mccartyi* was the sole organohalide-respiring microorganism and represented 75% of the total bacteria [49]. Then, the culture was operated as a batch-fed system in a sequential manner (i.e., fill-and-draw bioreactors) at an HRT of 30 days in a 240 mL serum bottle, including 160 mL liquid phase and 80 mL headspace [50]. Briefly, every 10 days, 50 mL of culture was withdrawn and anaerobically replaced by the same volume of fresh mineral medium (1 g/L NaCl, 0.048 g/L Na_2_S, 2.52 g/L NaHCO_3_, 0.3 g/L NH_4_Cl, 0.2 g/L KH_2_PO_4_, 0.5 g/L MgCl_2_·6H_2_O, 0.015 g/L CaCl_2_·2H_2_O, and 1 mL/L of metal solution and 10 mL/L of vitamin solution) [51]. The culture was then fluxed with a mixture of N_2_/CO_2_ (70:30) to remove the RD’s byproducts, fed with TCE (0.5 mM), and supplied with lactate (0.5 mM) as a fermentable electron donor. A volume of 200 mL of the dechlorinating enrichment culture at a pseudo-steady state (i.e., after three HRTs) was used as an inoculum for the fixed bed reactors (see below). An aliquot was also sampled for the biomolecular analysis, including the characterization of the microbiome composition (see below).

### 2.4. Analytical Methods and Calculations

#### 2.4.1. Chlorinated Aliphatic Hydrocarbon (CAH) Determination

The disappearance of TCE and the formation of RD products (cis-DCE, VC, ethylene) was monitored by taking 50 μL of the gas phase with a Hamilton^®^ Gastight SampleLock syringe (Merck, Darmstadt, Germany), injected directly into a Dani Master Gas Chromatograph (DANI Instruments, Contone, Switzerland) with a flame ionized detector (GC-FID), and a glass column with a phase 4% Carbowax 20 M, matrix 80/120 Carbopack B DA support (Merck KGaA, Darmstadt, Germany). The carrier gas was He (purity level: ≥99.998%) at 25 mL/min, the injector’s temperature was 200 °C, the oven temperatures were 50 °C for 2 min, 20 °C/min, and 210 °C for 5 min; the FID detector temperature was 200 °C with 25, 35, and 220 mL/min as N_2_-H_2_-air flows, respectively. The compounds revealed from the gas analysis were expressed in nominal concentration (C_N_), which refers to the concentration of the measured compound as if it were entirely present in the liquid phase (taking into account the relationship between gas and liquid concentration, according to the Henry’s law constant [52]). Quantification was possible with a calibration line. The limit of detection was 58 µM for TCE, 170 µM for cis-DCE, 50 µM for VC, and 10 µM for ethylene. TCE IN was determined by analyzing the liquid phase sampled by the feeding solution with a DANI MASTER GC equipped with a DANI 86.50 headspace auto-sampler (DANI Instruments, Contone, Switzerland) and a TRB624 capillary column measuring 30 m × 0.53 mm ID × 3 um; a flame ionization detector (FID) was also used. The method was described in detail in Rossi et al., 2021 [46].

#### 2.4.2. Tracer Determination

Bromide (C_0_ = 100 mg/L ${\mathrm{Br}}^{-}$) was revealed with a Dionex ICS-1000 IC ion chromatograph (Sunnyvale, CA, USA), with a conductivity cell detector equipped with a Dionex AS-40 Autosampler, a Dionex IonPac™ AG14 precolumn (4 × 50 mm), and a Dionex IonPac™ AS14 IC column with a 4 mm AESR 500 suppressor (Thermo Fisher Scientific, Chelmsford, MA, USA). The eluent phase was prepared with 3.5 mM Na_2_CO_3_ and 1.0 mM NaHCO_3_, with 1.2 mL/min as the flow rate. On the other hand, for the test performed with NaCl (C_0_ = 0.02 M), a HandyLab^®^ 330 conductometer (SI Analytics, Weilheim, Germany) was used to measure the conductivity variation. The data were processed by drawing the F curve (C/C_0_) against time.

#### 2.4.3. Calculations

The TCE removal (%) was calculated as reported in Equation (1). Additionally, the percentage of the converted TCE in RD daughter products, here named “mass balance”, was calculated according to Equation (2), as loss mechanisms (i.e., adsorption, volatilization) and experimental errors may affect the result. This parameter indicated how much of the incoming TCE was converted into reduction products for the RD biological pathway only. Both parameters were calculated on the average of the running period and when the reactor reached a pseudo-steady state.

TCE removal (%) = TCE_in–_TCE_out_/TCE_in_ × 100 (1)

Mass Balance (%) = ∑([cisDCE] + [VC] + [ETH])/(TCE_in–_TCE_out_) × 100 (2)

The reaction rate (rRD) of the TCE-to-ethylene culture was calculated before the beginning of the test. In Equation (3), the numbers 2, 4, 6 indicate the moles of electrons necessary for the formation of the chlorinated intermediates and ethylene starting from 1 mol of TCE; and [cisDCE], [VC], and [ETH] represent the C_N_ (mM) detected at the end of the cycle (10 days).

r RD (meq/Ld) = (2[cisDCE]) + (4[VC]) + (6 × [ETH])/days(3)

### 2.5. Biomolecular Analysis

#### 2.5.1. Sampling and DNA Extraction

A volume of 10 mL of the dechlorinating inoculum was collected with a sterile syringe and filtered on polycarbonate filters (pore size 0.22 μm, 47 mm diameter, Nuclepore) to harvest the biomass and was then used for the DNA extraction. At the end of the reactor operations, 1 g of silica sand from the BR and 1 g of biochar from the BBR were collected with a sterile spatula by opening the columns and were then used for the DNA extraction. DNA was extracted from all of the collected samples with a DNeasy PowerSoil Pro Kit soil DNA extraction kit (Qiagen, Italy) following the manufacturer’s instructions. Purified DNA from each sample was eluted in 100 μL sterile Milli-Q and stored at −20 °C for further analysis.

#### 2.5.2. 16S rRNA Gene Amplicon Sequencing and Bioinformatics

A total of 4 ng of DNA extracted from each sample was used for 16S rRNA gene amplicon sequencing. The amplicon library was prepared to target the V1–V3 variable regions, as previously described [53]. PCR reactions were performed in 25 μL total volume containing Phusion High Fidelity Master Mix (Thermo Fisher Scientific, Rodano, Italy) and 0.5 μM final concentration of the library adaptors with V1–V3 primers (bacteria 27F: 5′-AGAGTTTGATCCTGGCTCAG-3′ and 534R: 5′-ATTACCGCGGCTGCTGG-3′). Libraries were purified using the Agencourt AMPure XP beads protocol (Beckmann Coulter, Cassina de´Pecchi, Italy) and the concentration was measured with a Qubit 3.0 fluorometer (Thermo Fisher Scientific, Rodano, Italy). Purified libraries were pooled in equimolar concentrations and diluted to 4 nM. PhiX Control (15%) was added to the pooled libraries. Samples were paired-end sequenced (2 × 301 bp) on a MiSeq (Illumina, Milano, Italy) instrument using a MiSeq Reagent Kit v3, 600 cycles (Illumina) following the standard guidelines. NGS raw data were processed and analyzed using QIIME 2 software tools 2018.2 release and the DADA2 algorithm [54]. The taxonomic analysis was based on a Naive–Bayes classifier trained on 16S rRNA gene sequences clustered at 99% similarities within the Silva 132–99 database allowing for the construction of a data set of amplicon sequence variants (ASVs). Silva 132–99 database available on line: https://www.arb-silva.de/documentation/release-132/ (accessed on 13 December 2017).

Raw sequence reads were deposited in the DDBJ/ENA/GenBank under the BioProject PRJNA772109 (BioSample accessions: SAMN22365179, SAMN22365180, SAMN22365181).

#### 2.5.3. 16S rRNA Gene Amplicon Sequencing and Bioinformatics

The abundance of *D. mccartyi* species was estimated via real-time PCR (qPCR) through the quantification of the 16S rRNA gene copy numbers. The reductive dehalogenase genes linked to the TCE-to-ethylene dechlorination (i.e., *tceA, bvcA*, *vcrA*) were also quantified by employing the absolute quantification method with TaqMan^®^ chemistry [55]. Reactions were conducted in triplicate in a CFX96 Touch^TM^ Real-Time PCR Detection System (Bio-Rad, Segrate, Italy) in 20 µL total volume, including SsoAdvanced^TM^ Universal Probes Supermix (Bio-Rad, Segrate, Italy), 3 μL of DNA as a template, and 300 nM of each primer/probe (sequences reported in Ritalahti et al., 2006 [55]). Standard curves for the absolute quantification were constructed by using the long amplicons method previously reported [56]. Quantitative data were reported as gene copy numbers/L of TCE culture or per gram of silica sand or biochar.

## 3. Results and Discussion

### 3.1. TCE-to-Ethylene Inoculum: Kinetic Performance and Characterization

**Figure 2:**
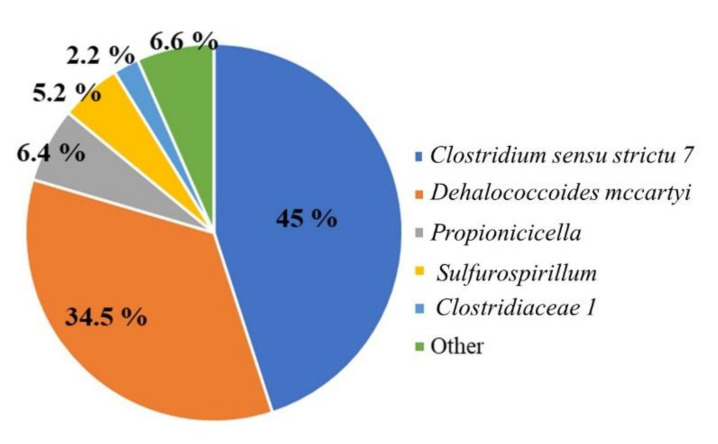
The 16S rRNA gene amplicon sequencing of the dechlorinating inoculum.

The TCE dechlorinating culture used as an inoculum for the column reactor was capable of complete TCE biological removal up to ethylene, already after 10 days from the feeding, with an RD rate of 0.34 ± 0.06 meq/Ld. According to the dechlorinating performances, the inoculum culture was characterized by an enriched microbiome with *D. mccartyi* (34.5%) as the sole organohalide-respiring microorganism (Figure 2). In detail, *D. mccartyi* 16S rRNA accounted for 4 × 10^8^ gene copies/L of culture. Among the reductive dehalogenase tested, *tceA* and *bvcA* were also found (1.06 × 10^8^ and 9.26 × 10^5^ gene copies/L, respectively), while *vcrA* was detected at an abundance of ≤1 × 10^3^ gene copies/L (detection limit, d.l.). Additionally, the fermentative species *Clostridium sensu stricto 7* (45%) were dominant in the culture, according to the feeding with lactate as a fermentable organic substrate to supply electron donors for the RD.

### 3.2. Results from Tracer Tests

**Figure 3:**
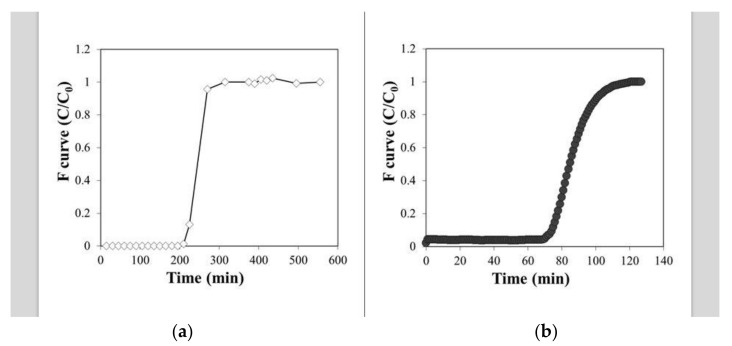
Tracer test curves for the BR (**a**) and BBR (**b**).

Step-signal experimental data are reported in Figure 3a,b. The HRT was calculated from the ratio between the geometric volume of the reactor and the flow rate of the test. The calculated porosity was 42% for the BR and 37% for the BBR (considerably lower, due to the presence of PWB). As a result, the pore volume was approximately 59 and 46 cm^3^ for the BR and BBR, respectively.

### 3.3. The BR Monitoring: The RD Mechanism 

**Figure 4:**
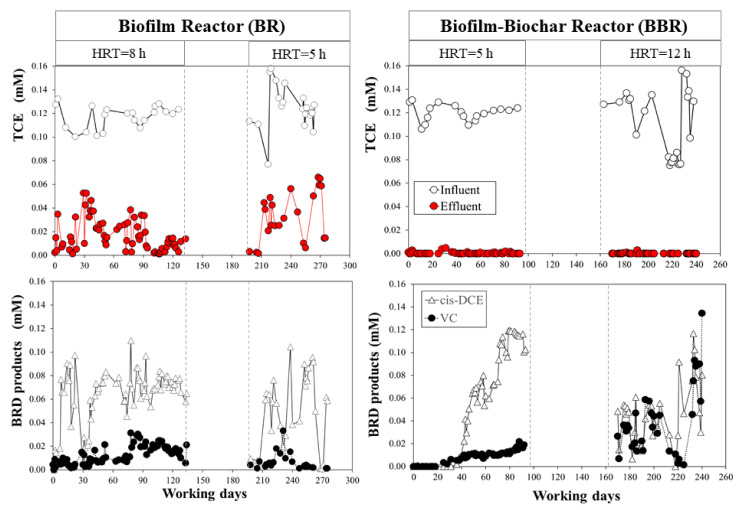
Reactor monitoring. The interruption period corresponds to the suspension of activities due to the COVID-19 health emergency in the city of Rome.

The BR worked continuously for 134 days at 0.36 L/d (run I condition). From the beginning of the test, the activity of the biofilm was revealed from the presence of the daughter products in the outlet current (Figure 4). With 3.4 h of effective residence time, cis-DCE was observed as the dominant product of the RD, with an average concentration of about 0.065 ± 0.021 mM, and 0.014 ± 0.008 mM of VC. However, the parental compound was not completely biodegraded; thus, TCE was detected in the effluent. The average TCE in the outlet was 0.016 ± 0.014, while the average TCE in the influent was 0.11 ± 0.01 mM. The average of TCE removal (%) was 86.9 ± 11.9, calculated according to Equation (1). Nevertheless, the mass balance % (Equation (2)) was about 77.15 ± 22.95%, indicating that TCE was mainly removed by the RD mechanism, unless there were sampling errors or leakage of gaseous phases. The total volume of contaminated solution treated during run I was 47 L (corresponding to 790 numbers of BV). Run II (77 days of operation) shows a behavior not very different from the previous one, but a dependence by a reduction of the residence time was highlighted.

As mentioned above in Table 1, the HRT was 5 h, corresponding to an effective residence time of 2 h (with a daily flow rate of 0.6 L/d). In this case, the daughter product concentrations confirmed a lower efficiency of the biofilm in the TCE degradation as the TCE_out_ increased, with an average of 0.032 ± 0.021 mM. Indeed, a lower concentration was revealed both for cis-DCE and VC, with 0.049 ± 0.032 mM and 0.006 ± 0.007 mM, respectively. The average TCE removal (%) was 78.73 ± 19.79%, calculated on the second run period, which was lower than the first run. The hypothesis is that the worst degradation capacity of TCE is due to a decrease of HRT and, therefore, of biological kinetics. In this regard, after only 24 h, even the TCE-to-ethylene batch culture from which the reactor inoculation came still clearly showed the presence of the cis-DCE and VC intermediates. In the investigated flow-rate range, we maintained a laminar flow regime and, therefore, the mass transfer was not affected by the change in bulk velocity. Furthermore, the mass balance calculated decreased to 56.9 ± 31.8%, probably due to systematic and random errors. The second run lasted 77 days, corresponding to 615 numbers of BV (close to 36 L treated). Overall, the RD mechanism alone did not lead to a quantitative conversion of the TCE, and this was even more evident in the test performed with a higher flow rate and lower residence time (run II).

### 3.4. The BBR Monitoring: The CAB Mechanism 

**Figure 5:**
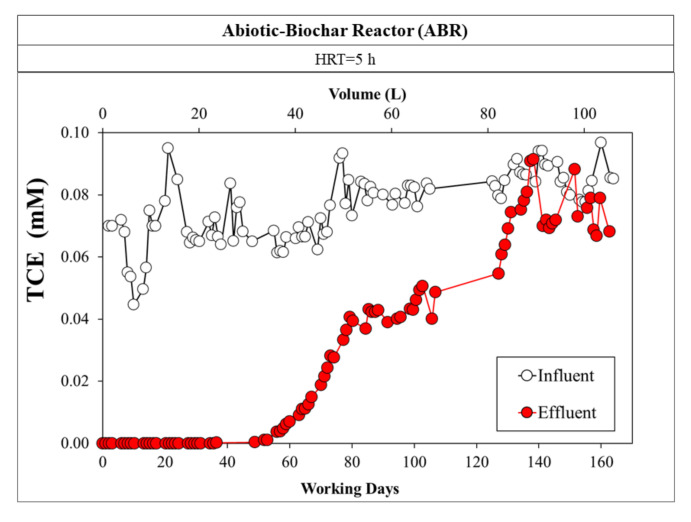
ABR monitoring represents the only adsorption mechanism of TCE onto the PWB.

The data trend for the BBR showed the copresence of the TCE-removal mechanisms (Figure 4). In particular, the first month of run I reported that adsorption was certainly the dominant mechanism, and neither TCE nor its reduction products were detected for almost 300 numbers of BV. Comparison with the ABR (Figure 5), working at the same HRT (5 h) and average input concentration of TCE (0.08 ± 0.01 mM), clearly demonstrated that, in the absence of the biological mechanism, the adsorbent zone of PWB reached a breakthrough point with a consequent increase in the concentration of TCE in the effluent [46,57]. Thereafter, the cis-DCE concentration began to rise slowly, accompanied by VC, suggesting that the less-chlorinated intermediates were not likely adsorbed and moved differently in the BBR’s reactive zone. The TCE removal calculated in this period was 99.6 ± 0.79%. On the other hand, the mass balance % was not calculated because cis-DCE and VC concentrations in the effluent were a function of time; thus, a steady-state condition was not reached. This test was performed for 93 days (1245 numbers of BV), with 53 L of treated water with no parental compound detected in the effluent.

Indeed, molecules of different sizes and hydrophily are involved in multicomponent adsorption in combination with biological reactions [57], hence the chlorinated compounds were retained by the PWB with a different affinity. As a result, highly chlorinated compounds predominate over reducing products at adsorption sites. This behavior was confirmed by the isotherm curve of cis-DCE onto the PWB, which showed a lower affinity with respect to TCE, in the range of 0–120 mg/L of equilibrium concentration investigated (with a maximum of 47.74 ± 7.05 mg cis-DCE/g PWB and 0.0286 ± 0.0084 L/mg as a Langmuir constant). Therefore, the TCE would have been adsorbed primarily in the early zones of the biochar bed, whereas a stratigraphic effect would occur in the rest of the reactor.

After the COVID-19 lockdown in the city of Rome (Italy), run II (with 5 h of effective residence time) was monitored for 70 days (355 BVs) with a TCE average concentration of the feeding solution of about 0.10 ± 0.03 mM. As shown in Figure 4, a decrease in the incoming TCE concentration occurred in the 220 to 240-day period, due to an error when preparing the feed solution. At the outlet, only the reduction products were detected, while TCE was always below the detection limit. At the end of the test, the average of cis-DCE was 0.047 ± 0.027 mM, with 0.040 ± 0.030 mM of VC. As a result, while in the first run the relevant product was the cis-DCE, the higher residence time of run II may have stimulated the metabolism through the second step of conversion (cis-DCE to VC). This phenomenon was even more evident during the last days of the test, where VC was the prevalent compound revealed. Moreover, ethylene was detected in trace (µM order of magnitude), even if not constantly (data not shown). The calculated average of TCE removal was about 99.87 ± 0.51%, and the mass balance during this period was about 82.79 ± 49.44%, suggesting that the dominant mechanism was the biological one. The high value of the standard deviation is linked to the fact that the sum of cis-DCE and VC on some days exceeded the mM of TCE entering (giving values higher than 100%). Overestimation of gas concentration (likely accumulation in the sampling cell with increased pressure) and/or degradation of adsorbed TCE are possible phenomena. The total volume treated during run II was 16.5 L.

Overall results suggest that, in the BBR reactor, a synergy between the phenomenon of multicomponent adsorption and biological metabolism was established, and these hypotheses are consistent with recent investigations. Particularly, Liu et al. highlight that reducing dissolved contaminant concentrations by immobilization on biochar create an environment that is less toxic to the biofilm, allowing it to survive even at high contamination loads [58]. These findings suggest that the activity of the biofilm could also have increased the adsorbent efficiency of the PWB, since the breakthrough point for the TCE was not observed. An “adsorption regeneration” process was proposed by the study of Cheng and colleagues, where the biologic degradation of adsorbed-nonylphenol on biochar was described [37]. These results support the synergistic effect of the CAB process and open doors to a possible prolonged lifespan of the adsorbent material.

### 3.5. Reactor′s Microbial Community Characterization

**Figure 6:**
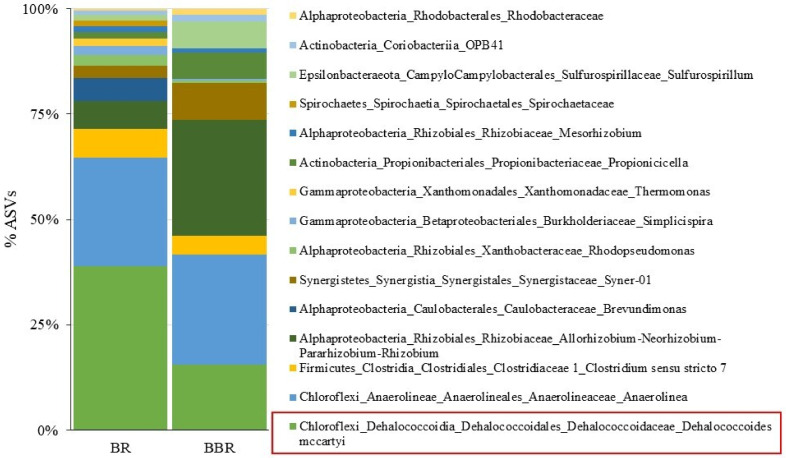
Microbiome composition of the BR and the BBR evaluated via 16S rRNA gene amplicon sequencing.

In line with the kinetic performances of the system, the bacterial composition of the BR and BBR showed the occurrence of *D. mccartyi* as the dominant microorganism in both compartments. One 16S rRNA gene sequence affiliated with *D. mccartyi* (100% similarity with strain 195 by Blast alignment) was found as the most abundant at the end of the reactor operations (Figure 6).

The same sequence was previously found in the TCE dechlorinating inoculum used for the start-up of the system (BioSample accession number: SAMN22365179), suggesting that the start-up of the reactor with the dechlorinating enrichment allowed the establishment of the actively growing dechlorinating biomass. In detail, *D. mccartyi* dominated the BR representing 30.7% of the total ASVs, with an absolute abundance of 4.5 × 10^5^ *D. mccartyi* 16S rRNA gene copies/g silica sand. Among the reductive dehalogenase genes analyzed, 1.4 × 10^5^ gene copies/g of *tceA* were found, while *bvcA* and *vcrA* genes were lower than the detection limit (≤1 × 10^3^ gene copies/g). Additionally, fermentative bacteria were also found, including members of the family *Clostridiaceae* (genus *Clostridium*), according to the feeding with lactate as a fermentable organic substrate acting as the electron donor for the reductive dechlorination [59].

Similarly, *D. mccartyi* was also established in the BBR compartment, representing 13% of the microbial community with an absolute abundance of 9.2 × 10^5^ *D. mccartyi* 16S rRNA gene copies/g of biochar. The gene *tceA* was the most abundant reductive dehalogenase gene found (8.16 × 10^6^ gene copies/g), while *bvcA* and *vcrA* were found to a minor extent (1.28 × 10^5^ and 8.01 × 10^3^ gene copies/g, respectively). The BBR was also characterized by the presence of genera belonging to the family *Rhizobiaceae (Alphaproteobacteria)*, including *Allorhizobium-Neorhizobium-Pararhizobium-Rhizobium*. This finding is in line with the origin of the biomaterial used in the BBR. Indeed, bacteria of the family *Rhizobiaceae* are usually soil-borne and found in association with plant roots, where they mostly rely on a saprophytic lifestyle, degrading soil organic compounds and plant exudates, and are often isolated due to their association with plants [60].

Interestingly, even if *D. mccartyi* was detected in both of the reactors at the end of the experiment, the *tceA* gene was found at relevant abundance only in the BR, coding for the TceA dehalogenase involved in the TCE-to-VC dechlorination. Conversely, in the BBR, both *bvcA* and *vcrA* genes were found according to kinetic trends of the reactors, coding for the BvcA and the VcrA dehalogenases involved in the cis-DCE/VC-to-ethylene dechlorination.

It is possible that the higher absolute abundance of *D. mccartyi* in the BBR compared to the BR was probably due to the microporous structure of PWB, which may create suitable habitat for the adhesion of microorganisms [58] and also may provide more nutrients or electrons available to stimulate bacterial growth as a result of its porous property [61]. Our results are in strong agreement with those reported by Chen and coworkers (2018), in which an increase in the relative abundances of the genera *Dehalococcoides* and *Dehalogenimonas* was observed in the microcosm with a high biochar dosage (the reaction investigated was the reductive debromination of 2,2′,4,4′-tetrabromodiphenyl ether) [61].

To the best of our knowledge, this is the first study reporting the occurrence of *D. mccartyi* species to remove TCE on biochar from wood waste, and a positive effect of biochar due to its electron transfer capacity should not be excluded [33,39,62].

These results enable the feasibility of this material as a suitable candidate, under optimized conditions, to support bioreducing dechlorination processes in engineered systems. Undoubtedly, the flow rate and length of the reactive zone must be considered to optimize the reactor’s design and avoid VC accumulation, since its reduction remains the bottleneck step in the biological dechlorination reaction [17,63].

## 4. Conclusions

The CAB mechanism was proposed as a promising strategy to manage contamination at the level of secondary sources/areas with a higher concentration of contaminants. The two synergistic mechanisms make it possible to immobilize and reduce the spread of contamination (due to a rapid adsorption kinetic) and to biodegrade, thanks to the addition of an electron donor. In this study, the experiment performed in a fixed-bed column allowed a simulation of a condition close to a permeable reactive barrier (PRB) or an external bioreactor (with a reactive area consisting of biochar and *D. mccartyi*-biofilm). In parallel, two reactors with the same geometry were made to simulate the individual mechanisms separately (the BR—only biodegradation, and the ABR—adsorption only).

The reactors worked continuously, despite two months of COVID-19 emergency lockdown in the city of Rome not allowing the monitoring of the effluent. However, they were continuously fed with an average TCE per day of between 3 and 4 mg/day. The BBR maintained good performance in both of the flow rate configurations and for the entire duration of the experiment (8 months). The results are encouraging for several reasons:

1. The PWB had a positive effect on the development and growth of a specific dechlorinating community;
2. The BBR showed good stability and long-lasting adsorbent efficacy related to ABR;
3. The CAB process should allow working under conditions that would normally exclude biological treatment as the only removal mechanism (e.g., low residence times and high pore water velocity);
4. The PWB is a cost-free material that has not undergone an activation process.

Moreover, the study demonstrates a different affinity of the less chlorinated intermediates for the adsorbent material (depending on the hydrophobicity of the molecule); hence, the lower affinity of the VC must be considered and kept under control.

This study suggests that biochar is a promising candidate to be employed in engineered systems for the in situ reductive dechlorination treatments, under the principles of the circular economy. This approach should enable the use of a byproduct of an energy recovery process as part of the environmental matrix requalification. For the use of biochar to be sustainable and conscious on a large scale, research should concentrate on biochar derived from waste, reducing environmental impact and costs, and establishing quality standards for safe use in all ecosystems. Future research should investigate biochar–bacterial cooperation in electron transfer capacity, which is an important factor in redox pathways.

## Figures and Tables

**Table 1:** Experimental operating conditions of the BR and BBR.

	“Biofilm Reactor” (BR)		“Biofilm–Biochar Reactor” (BBR)	
	Run I	Run II	Run I	Run II
Flow rate (L/d)	0.36 ± 0.08	0.6 ± 0.02	0.6 ± 0.01	0.25 ± 0.12
HRT (h)	8	5	5	12
Effective Residential Time (h)	3.4	2	1.6	3.8
Pore Water Velocity (cm/d)	163	272	352	147
